# Chronic helminth infection does not impair immune response to malaria transmission blocking vaccine Pfs230D1-EPA/Alhydrogel® in mice

**DOI:** 10.1016/j.vaccine.2019.01.027

**Published:** 2019-02-14

**Authors:** Camila H. Coelho, Pedro Henrique Gazzinelli-Guimaraes, Jennifer Howard, Emma Barnafo, Nada A.H. Alani, Olga Muratova, Ashley McCormack, Emily Kelnhofer, Joseph F. Urban, David L. Narum, Charles Anderson, Jean Langhorne, Thomas B. Nutman, Patrick E. Duffy

**Affiliations:** a Laboratory of Malaria Immunology and Vaccinology, National Institute of Allergy and Infectious Diseases, National Institutes of Health, Rockville, MD, USA; b Laboratory of Parasitic Diseases, National Institute of Allergy and Infectious Diseases, National Institutes of Health, Bethesda, MD, USA; c US Department of Agriculture, Agricultural Research Service, Beltsville Human Nutrition Research Center, Diet, Genomic and Immunology Laboratory, Beltsville, MD, USA; d The Francis Crick Institute, London, UK

**Keywords:** Malaria transmission blocking vaccine, *H. polygyrus* bakeri, Pfs230, *P. falciparum*, Helminth intestinal infection, Immunoregulation

## Abstract

**Introduction:**

Malaria transmission blocking vaccines (TBV) are innovative approaches that aim to induce immunity in humans against *Plasmodium* during mosquito stage, neutralizing the capacity of the infected vectors to transmit malaria. Pfs230D1-EPA/Alhydrogel®, a promising protein-protein conjugate malaria TBV, is currently being tested in human clinical trials in areas where *P. falciparum* malaria is coendemic with helminth parasites. Helminths are complex metazoans that share the master capacity to downregulate the host immune response towards themselves and also to bystander antigens, including vaccines. However, it is not known whether the activity of a protein-based malaria TBV may be affected by a chronic helminth infection.

**Methods:**

Using an experimental murine model for a chronic helminth infection (*Heligmosomoides polygyrus bakeri - Hpb)*, we evaluated whether prior infection alters the activity of Pfs230D1-EPA/Alhydrogel® TBV in mice.

**Results:**

After establishment of a chronic infection, characterized by a marked increase of parasite antigen-specific IgG1, IgA and IgE antibody responses, concomitant with an increase of systemic IL-10, IL-5 and IL-6 levels, the Hpb-infected mice were immunized with Pfs230D1-EPA/Alhydrogel® and the vaccine-specific immune response was compared with that in non-infected immunized mice. TBV immunizations induced an elevated vaccine specific-antibody response, however Pfs230D1 specific-IgG levels were similar between infected and uninfected mice at days 15, 25 and 35 post-vaccination. Absolute numbers of Pfs230D1-activated B cells generated in response to the vaccine were also similar among the vaccinated groups. Finally, vaccine activity assessed by reduction of oocyst number in *P. falciparum* infected mosquitoes was similar between Hpb-infected and immunized mice with non-infected immunized mice.

**Conclusion:**

Pfs230D1-EPA/Alhydrogel® efficacy is not impaired by a chronic helminth infection in mice.

## Highlights

• Pfs230 is a candidate malaria transmission blocking vaccine against *P. falciparum.*
• Pfs230 vaccine is being tested in areas where malaria and helminth infections are co-endemic.
• Chronic helminth infection induces a marked increase in systemic Th2 and regulatory cytokine levels in mice.
• Chronic *H. polygyrus bakeri* infection does not alter Pfs230 vaccine specific-antibody levels.
• Functional activity of Pfs230 vaccine was not impaired by chronic helminth infection in mice.

## 1 Introduction

To eradicate malaria, novel tools must integrate with existing approaches to reduce parasite transmission. One promising potential tool is a transmission-blocking vaccine (TBV), which aims to halt transmission by inducing antibodies targeting antigens expressed by the parasite in the mosquito host [5], [32]. Pfs230, a protein present on the surface of *Plasmodium falciparum* gametes, is a leading candidate for a TBV.

Recently, a recombinant form of the first 6-cysteine rich domain of Pfs230 (domain 1, D1) was produced with the quality characteristics and quantity suitable for human clinical trials using the *Pichia pastoris* expression system [14]. In order to enhance immunogenicity, the ∼20 kDa recombinant Pfs230D1 protein was chemically conjugated to a carrier protein (ExoProtein A, EPA) and formulated in an adjuvant (Alhydrogel®). This vaccine candidate is currently in clinical trials in endemic areas [6].

Malaria-affected areas are often co-endemic with helminth parasite infections. Helminth parasites belong to multiple taxonomic groups, but collectively they share the capacity to downregulate the parasite-directed host immune response [12], [22], [16], [10]. During chronic infection, helminths modulate immune responses to bystander pathogens [17], [2], and to some vaccine antigens [24], [7].

The cytokine response to most helminth parasites (including the gastrointestinal nematode parasites) is characteristically both Th2- and IL10-dominated; the IL-10 response appears to derive from both adaptive (aTreg) and natural T regulatory cells (nTreg) [33], [18]. These prototypical responses driven by helminths or helminth-derived molecules have been shown to alter the responses to some types of vaccines [26], [1], though this is not a universal finding [9], [30].

To date, few studies have examined whether a malaria TBV can be modulated by infection with intestinal helminth parasites. It has been recently suggested that *Heligmosomoides polygyrus bakeri* (Hpb) infection impairs the immunogenicity of a *Plasmodium falciparum* (Pfs25) DNA TBV, although this infection did not impair immunity to irradiated sporozoites [21].

Hpb is a natural intestinal parasite of mice, capable of establishing long-term chronic infections in many strains of mice which is ideally suited for lengthy immunization studies. During the infection, Hpb induces a markedly polarized early Th2 response characterized by increased IL-4, IL-13 and IgE production [23]. However, this persistent type 2 response shifts to long-lasting chronic infection, characterized by a strong regulatory response with expanded frequency of regulatory T cells and production of IL-10, peaking at day 28 post-infection [8]. At this stage of infection, the ability of Hpb to down-modulate responses to unrelated bystander antigens, including vaccine candidates, has been extensively demonstrated [31], [27], [25], [29], [21].

In this context, we used the mouse model of intestinal infection with Hpb to assess whether transmission-blocking immunity induced by Pfs230D1-EPA/Alhydrogel® would be impaired by helminth infection. Our findings demonstrate that chronic Hpb infection does not affect antibody responses or transmission-blocking activity induced by Pfs230D1-EPA/Alhydrogel® immunization. This supports the feasibility of TBV use in areas where intestinal helminths and malaria are co-endemic.

## 2 Materials and methods

### 2.1 Ethics statement

All animals were infected, vaccinated and sampled according to protocols approved by the NIAID Animal Care and Use Committee (Protocol #LPD-6).

### 2.2 *H. polygyrus bakeri* infection in mice

For each experiment, 10 BALB/c mice per group (male, 6 weeks old, Taconic Farm, USA) were infected with 200 *H. polygyrus bakeri* (Hpb) infective larvae (L3) by oral gavage 28 days before the first dose of the Pfs230D1-EPA/Alhydrogel® vaccine. The confirmation of Hpb infection and intensity follow-up were determined by fecal egg counts at days 25, 53 and 63 post-infection using standard protocols [4].

### 2.3 *H. Polygyrus bakeri* excretory/secretory (HES) antigen preparation

HES antigens from adult worms were prepared as described by Johnston et al. [13] with some minor modifications. Briefly, Hpb adult worms were isolated from the duodenum of BALB/c mice inoculated 14 days earlier with 200 infective 3rd stage larvae (L3). The worms were soaked and washed six times in Hanks' Solution (supplemented with 5 U/ml penicillin and 5 μg/ml streptomycin) and then placed in RPMI 1640 culture media plus a standard antibiotic mixture of penicillin (5 U/ml), streptomycin (5 μg/ml) and gentamicin (1%), distributed at approximately 400 adult worms per 2 ml in 24-well culture plates for 1–2 week. HES-containing culture media were collected at intervals of twice per week, and then were pooled out and concentrated over a 3000 MWCO filter. The protein concentration was determined by Bradford assay and the HES were used for the ELISA assays to measure helminth specific antibody response.

### 2.4 Pfs230D1-EPA alhydrogel® vaccine preparation

Pfs230D1-EPA (lot MV-1721) is a conjugate produced by chemically cross-linking Pfs230D1, a highly purified recombinant protein corresponding to Pfs230 expressed by gametocytes and on gametes, to rEPA, a highly purified recombinant protein corresponding to a mutant and detoxified Exoprotein A from *Pseudomonas aeruginosa*. The conjugate Pfs230D1-EPA (MV-1721) is composed of 54.6% Pfs230D1 and 45.4% EPA and manufactured under current good manufacturing practice (cGMP) with methods developed at the Laboratory of Malaria Immunology and Vaccinology (LMIV), National Institute of Allergy and Infectious Diseases, National Institutes of Health as described previously [14].

Pfs230D1-EPA (MV-1721) was formulated by adding 8.2 µL of the vaccine aseptically to each of two sterile Wheaton glass vials containing a mixture of 359.8 µL of Phosphate Buffered Saline (PBS, 1X, Gibco Catalog #10010-023) and 32 µL of Alhydrogel® 2% (Aluminum Hydroxide Gel Adjuvant, Brenntag Biosector, Batch #5240, EXP Dec 2017, Aluminum content: 9.8 mg/mL). Mixing was done using a Rotamix Rotator (ATR model #10101) at 16–24 rpm for 60 min, at room temperature (RT). The final formulation was then stored at 2–8 °C for approximately 24 h prior to use for immunization of the mice.

### 2.5 Mouse immunization with Pfs230D1-EPA/Alhydrogel

Mice were infected with *H. polygyrus bakeri* as described above at day −28. After 28 days of infection with Hpb, ten BALB/c mice were immunized intramuscularly in the leg, with 1 µg of Pfs230D1-EPA/Alhydrogel® in 50 uL of PBS using a “hubless” syringe. The vaccine immunization day was considered as day 0. The second dose was given 28 days after the first vaccine dose (day 28). On day 35, 7 days after the second dose, all mice were euthanized. Experiments were performed in two independent sets of infection and immunizations comprising 5 mice per group.

### 2.6 Sample collection

**Fig. 1:**
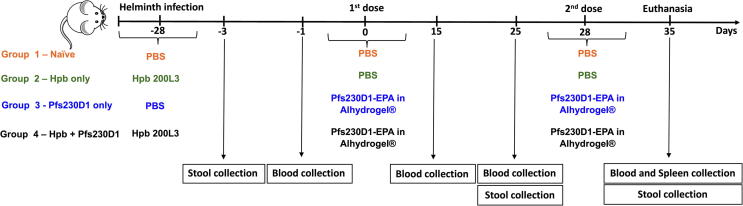
Immunization strategy for Pfs230D1-EPA/Alhydrogel® in mice previously infected (or not infected) with *H. polygyrus bakeri*. After 28 days of infection with 200 infective Larvae (L3i) of *H. polygyrus bakeri*, BALB/c mice (n = 10 for each group) were vaccinated intramuscularly with two doses of 1 µg of Pfs230D1-EPA. Blood was collected at days −1, 15, 25 and 35.

Retro-orbital blood was collected from mice at days 0, 15 and 25 of the study (Fig. 1). Stool samples were collected at days −25, 25 and 35. On day 35, mice were euthanized, exsanguinated, and spleens were removed for isolation of B cells.

### 2.7 Detection of Pfs230D1 antibody response in sera

Antibody responses to Pfs230D1 were measured using an enzyme-linked immunosorbent assay (ELISA). Immulon® 4HBX plates were coated with 1ug/well of recombinant Pfs230D1. Plates were incubated overnight at 4 °C and blocked with 320 µL of buffer containing 5% skim milk powder in Tris-buffered saline for 2 h at RT. Plates were washed with Tween-TBS. Samples (dilution 1:500) were added to antigen-coated wells in triplicate and incubated for 2 h at RT. Plates were washed, then 100 µL of alkaline phosphatase labelled goat anti-mouse IgG, IgG1, IgG2a or IgG3 (KPL) were added and incubated for 2 h at RT. The plates were washed and a colorimetric substrate (p-nitrophenyl phosphate; Sigma) was added. Plates were read at absorbances of 450 nm and 550 nm on a multi-well reader (Molecular Devices).

### 2.8 Detection of anti-Hpb antibody response in sera

Antibody responses to Hpb were measured by ELISA. Immulon® 4HBX plates were coated with 1ug/well of Hpb adult worm excretory/secretory (HES) antigen. Plates were incubated overnight at 4 °C and, after washing, blocked with 200 µL of PBS-BSA 5% for 1 h at RT. Plates were washed six times with Tween-TBS. 50uL of mouse serum (1:500 for IgG1, IgG2a, IgG3; 1:10 for IgE and IgA in PBS-BSA 1%) were added to antigen-coated wells in duplicate and incubated for 1 h at RT. Plates were washed and then incubated separately with biotinylated rat anti-mouse IgG1, IgG2, IgG3, IgE and IgA (BD Pharmingen) (1:250 in PBS-BSA 1%) for 1 h at RT. Plates were washed and incubated with 50 μL of streptavidin conjugated with HPR (1:200 in PBS-BSA 1%) for 30 min at RT. After washing, TBM-substrate was added (50 μL/well) and incubated for ten minutes in the dark. 25 µL of 2 N H_2_SO_4_ was used to stop the reaction and plates were read at absorbances of 450 nm and 550 nm on a plate reader (Molecular Devices).

### 2.9 Multiplex assay for sera cytokines

Twenty-five microliters of each mouse serum were used to determine levels of IL-5, IL-6, IL-10, IL-13, TNF-a and IFN-y by magnetic beads using Luminex multiplex assay (Milliplex; Millipore) based on the manufacturer’s recommendations.

### 2.10 Isolation of Pfs230D1-specific B cells

For isolation of Pfs230D1-specific B cells from splenocytes, we developed a biotin-streptavidin tetramer prepared with Pfs230D1 recombinant protein expressed in *Pichia pastoris* [14]. Tetramer preparation was performed as previously described [28], [11]. Briefly, Pfs230D1 protein was biotinylated (EZ-link Sulfo-NHS-LC-Biotin, Thermo Fisher) and bound to streptavidin previously conjugated with fluorochrome PE (Prozyme). A decoy tetramer was generated using BSA and the fluorochromes PE and DL-594, to assure reduction of unspecific bindings. Splenocytes were isolated and incubated with 1 µM decoy tetramer and PBS containing 10% FBS and Fc Block (anti-CD16/32 clone 2.4G2), to inhibit unspecific binding of Fc receptor expressing cells), for 5 min at room temperature in the dark. Then, Pfs230D1-PE tetramer at 1 µM was added to the tube and incubated at 4 °C protected from light for 20 min. Cells were washed with PBS containing 10% FBS and incubated with anti-PE magnetic beads (Miltenyi Biotec) for 20 min. Four mL of PBS were added, and the suspension was passed over magnetized LS columns for elution of Pfs230D1-PE specific cells.

### 2.11 Flow cytometry

After enrichment with PE and Decoy Tetramers, splenocytes were stained with Zombie Violet live/dead, Alexa Fluor 700 anti-Gr-1 (clone RB6-8C5), Alexa Fluor 700 anti-CD3 (clone 17A2), Alexa Fluor 700 anti-F4/80 (clone BM8), APC-Cy7 anti-B220 (RA3-6B2), PE-Cy7 CD19 (clone 6D5) and PercP/Cy5.5 anti GL7 (clone GL7) conjugated antibodies from Biolegend. Pfs230D1-specific B cells were gated and non-Pfs230D1 B cells were excluded. Cells were analyzed using LSR II cytometer (BD Biosciences) and analysed using FlowJo V.10 (Tree Star).

### 2.12 Transmission reduction activity

Transmission reducing activity (TRA), determined by the reduction of *P. falciparum* oocyst burden in the mosquito midgut, was evaluated using standard membrane feeding assay (SMFA) [19]. Briefly, an in vitro 15-day culture of stage V *P. falciparum* gametocytes (NF54 line) was diluted with washed O+ RBCs and an AB+ serum pool (not heat-inactivated) from malaria-naïve volunteers (Interstate Blood Bank, Memphis, Tennessee) to achieve 0.07–0.9% concentration of Stage V gametocytes and a 50% hematocrit. For each sample, 200 μL of diluted culture was mixed with 60 μL of test sample (mouse serum pool diluted 1:2 with naïve AB+ human serum pool) and immediately fed to pre-starved (24–30 h) 3–8-day-old *Anopheles stephensi* (Nijmegen strain) mosquitoes using a Parafilm® membrane in a mosquito feeder, kept warm with a jacket with 40 °C circulating water. After feeding, mosquitoes were kept at 26 °C and 80% humidity conditions to allow parasites to develop. On Day 8 after the feed, mosquito midguts were dissected and stained with 0.05–0.1% mercurochrome solution in water for at least 20 min. Infectivity was measured by counting oocysts in at least 20 mosquitoes per sample. Pre-vaccination serum pools from the same mice was used as negative control. Each sample was tested in two independent SMFAs, and the two TRA values were averaged to obtain a single subject level TRA for a given time point.

### 2.13 Statistical analyses

Analyses were performed using data from two independent experiments. Statistical analyses for Pfs230D1 IgG measurement, specific B cells quantification by flow cytometry, and SMFA were performed by One-way ANOVA and corrected for multiple comparisons. Data from cytokine levels and *Hpb* IgG measurements were analysed using the Kruskal-Wallis test followed by Dunn's multiple comparisons test. Parasite quantification in the stool samples was analysed by the Mann-Whitney test.

## 3 Results

To investigate if chronic helminth infection could impair the immunogenicity and/or efficacy of a malaria TBV, we infected BALB/c mice with Hpb L3 by gavage and 28 days later immunized them intramuscularly with two doses of Pfs230D1-EPA/Alhydrogel® (Fig. 1).

### 3.1 Hpb burden and anti-Hpb titers are maintained during vaccination with Pfs230D1-EPA/Alhydrogel®

**Fig. 2:**
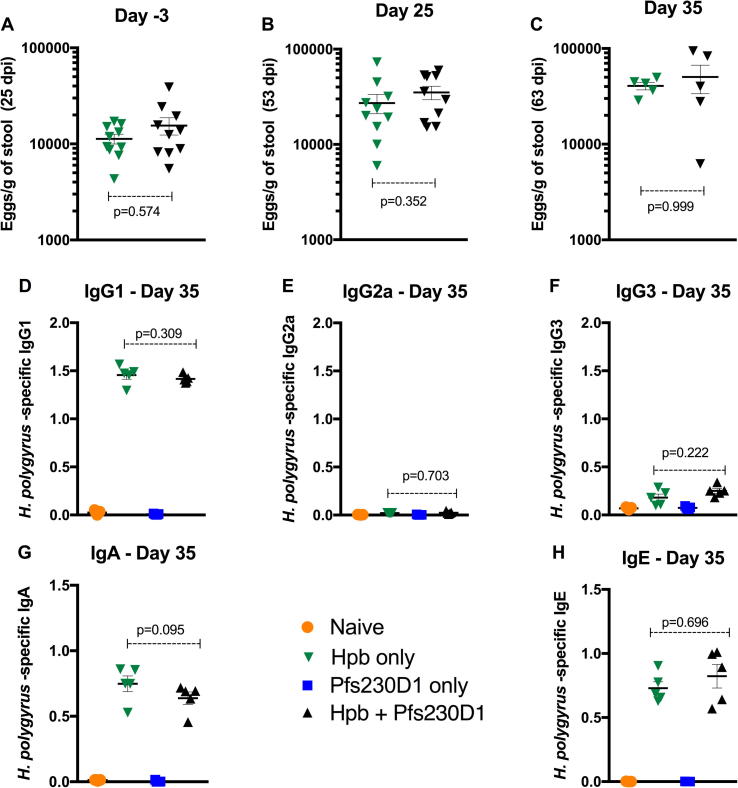
Hpb Infection pattern was sustained after immunization. (a) Amount of Hpb eggs were similar in stools collected in animals infected only or both infected/vaccinated at days −3 (p = 0.574), 25 (p = 0.841) and 35 (p = 0.999). (b) ELISA optical density in response to Hpb antigen in sera. Sera collected at days −1, 15, 25 and 35 were used to assess IgG1, IgG2a, IgG3, IgE and IgA responses to *H. polygyrus bakeri*. Data are shown as mean + SEM.

We assessed the parasite burden and chronicity of the helminth infection by quantification of the Hpb eggs in the stool at day 25 (3 day prior to immunization), day 53 (25 days after TBV immunization) and day 63 post-infection (35 days after TBV administration). We also assayed Hpb-specific antibody production at day 63 post-infection. At all 3 time points, all animals from group 2 and group 4 were infected with similar egg counts (Fig. 2A–C). Before the euthanasia at day 63dpi the parasite burden in both infected groups were the same (Day 63; 43,236 ± 3644 eggs/g of stool vs. 40,264 ± 16,674 eggs/g of stool, p > 0.900) (Fig. 2C). Similarly, levels of Hpb-specific antibodies did not differ between the 2 Hpb-infected groups of mice. The Hpb-specific antibody response for the 2 Hpb-infected groups was characterized by a highly significant increase in the IgG1 but not IgG2a levels at day 63 post-infection, when compared with group 1 (naïve controls) and group 3 (vaccinated but not Hpb-infected mice) (1.42 ± 0.04 OD and 1.45 ± 0.04 OD vs. 0.02 ± 0.01 OD and 0.01 ± 0.01 OD, p < 0.01, respectively) (Fig. 2D and E). No significant differences for IgG3 were seen among the four groups (Fig. 2F) and similar increases in IgA and IgE isotypes were observed in groups 2 and 4 compared to group 1 and 3 (Fig. 2G and H).

### 3.2 *H. polygyrus bakeri* infection does not alter antibody levels generated by Pfs230D1-EPA/Alhydrogel® vaccine

**Fig. 3:**
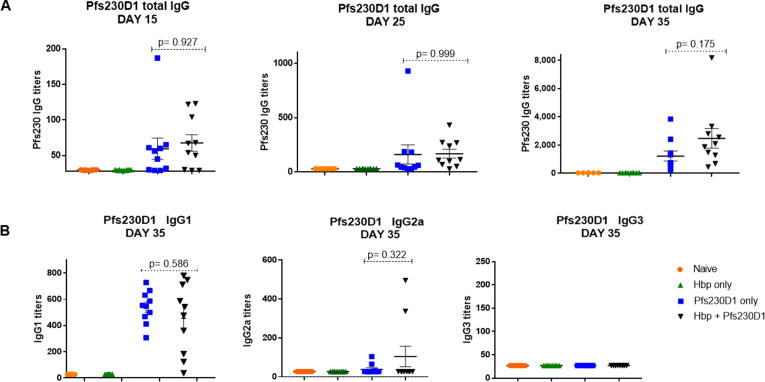
Pfs230D1 IgG titers in sera from mice. Mice were previously infected or not with *H. polygyrus baker,* and 28 days immunized or not with Pfs230D1-EPA/Alhydrogel® (n = 10 for each of the 4 groups, combined from two different immunization studies). Pfs230D1-specific antibodies generated with 1 µg of Pfs230D1 vaccine injected intramuscularly were measured by ELISA. Data are shown as mean + SEM.

We next measured levels of Pfs230D1-specific IgG in all groups of mice (Fig. 3). Pfs230D1 antibody levels were similar between infected and uninfected mice at day 15 (67.6 ± 36.8 units vs 59.4 ± 47.1 units p > 0.900) and at day 25 (168.2 ± 128.78 units vs. 161.3 ± 276.2 units, p > 0.900) post-vaccination. One week after the second vaccination (day 35), Pfs230D1 IgG titers were slightly higher in sera from Hpb-infected mice, but the difference was not statistically significant (2.469.5 ± 2198.8 units vs. 1220.2 ± 1123.4 units, p = 0.1756). Pfs230D1-specific IgG titers were not detected in sera from unvaccinated mice (groups 1 and 2). Pfs230D1-specific IgG1, IgG2a and IgG3 levels were measured at day 35. IgG1 levels were similar between groups that were Hpb-infected and vaccinated and those only receiving the TBV (455.9 ± 268.8 vs. 540.2 ± 124.6p = 0.5862). IgG2a and IgG3 levels did not differ between immunized mice and the naïve group (p > 0.900), nor between Hpb-infected/vaccinated mice and those only vaccinated (p = 0.322). Overall, these results indicate that chronic Hpb infection does not impact the IgG response to PfS230D1 immunization.

### 3.3 Pfs230D1 vaccination reduces serum levels of IL-10 but does not impact other cytokine profiles in Hpb-infected mice

**Fig. 4:**
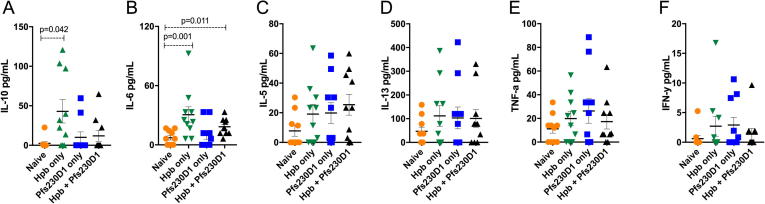
Serum cytokines from mice immunized with Pfs230D1-EPA/Alhydrogel® and previously infected with *H. polygyrus bakeri* (n = 10 for each group, determined in two different experiments). IL-10, TNF-a, IL-5, IL-13, IL-6 and IFN-y levels in sera were quantified by Luminex assay. Data are shown as mean + SEM.

At day 63 post inoculation (35 days following the first vaccination dose), Hpb infection induced a marked increase in IL-10 levels in the sera of group 2 compared to the uninfected animals (group 1; 42.9 ± 14.77 pg/mL vs. 2.4 ± 2.2 pg/mL, p = 0.042) or the uninfected but Pfs230-vaccinated animals (group 3; 10.1 ± 6.8 pg/mL). Interestingly, IL-10 levels did not increase in the animals of group 4 (Hpb-infected and Pfs230D1-vaccinated mice (11.9 ± 6.9 pg/mL) (Fig. 4A), as compared with group 2. Serum levels of IL-6 increased in both Hpb-infected group 2 (30.6 ± 7.9 pg/mL vs 7.3 ± 2.3 pg/mL, p = 0.001) and group 4 (18.4 ± 2.6 pg/mL vs 9.6 ± 4.2 pg/mL, p = 0.011) when compared to group 1 (Fig. 4B). Levels of TNF-alpha, IFN-gamma, IL-5 and IL-13 did not differ significantly.

### 3.4 *H. polygyrus bakeri* infection does not affect the number of activated B cells produced in Pfs230D1-EPA/Alhydrogel® vaccination

Supplementary data associated with this article can be found, in the online version, at https://doi.org/10.1016/j.vaccine.2019.01.027.

**Supplementary Fig. 1:**
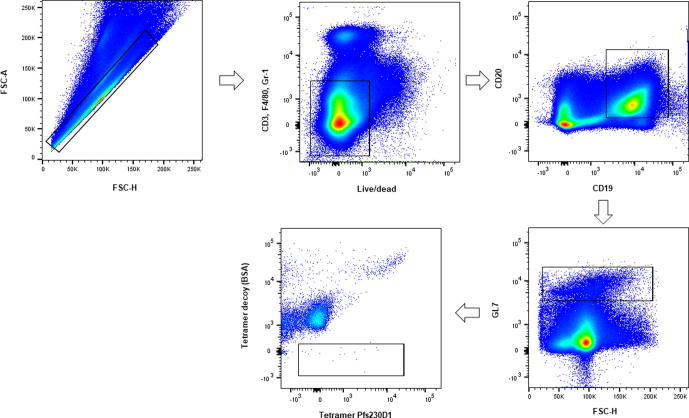

**Fig. 5:**
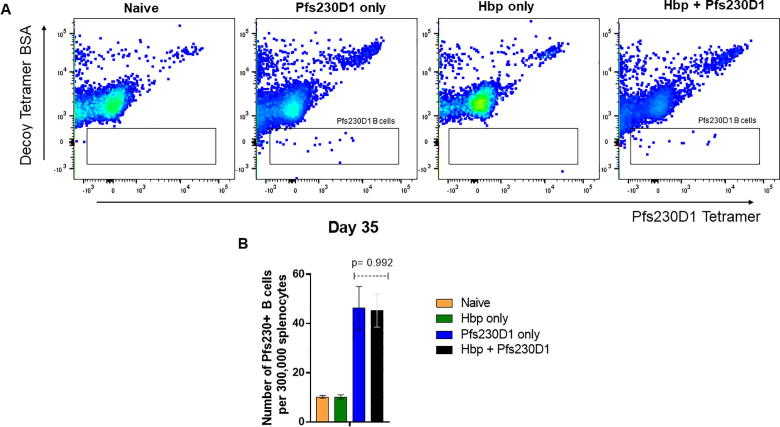
Pfs230D1-specific B cells identified in spleens from immunized mice (n = 5). (a) Identification of Pfs230D1B cells using a tetramer approach. Cells were gated in singlets, live, B220+, CD19+, GL7 + and after exclusion of unspecific binding by decoy antibody, cells were gated specifically in response to Pfs230D1 antigen, with the biotin-streptavidin tetramer expressing fluorochrome PE. Gating strategy is shown in Supplementary Fig. 1 (b) Frequency of Pfs230D1-specific B cells among 300,000 splenocytes.

To further characterize humoral responses and investigate whether Hpb infection affects vaccine-induced B cell generation, specific Pfs230D1-B cells were enumerated at day 35, seven days after the second immunization (Supplementary Fig. 1). The frequency of Pfs230D1-specific B cells was not statistically different between Hpb-infected and -uninfected groups that received the vaccine (45.2 ± 14.9 cells vs. 46.1 ± 19.2, p = 0.992, respectively) (Fig. 5).

### 3.5 Chronic helminth infection does not impair the functional activity of transmission blocking vaccine Pfs230D1-EPA/Alhydrogel®

**Fig. 6:**
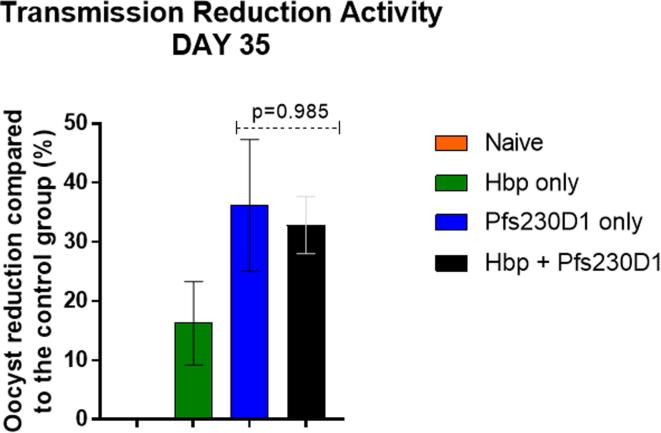
Standard Membrane Feeding assay with mouse sera to assess oocyst reduction after Pfs230D1 immunization and helminth infection. Sera from each experimental group was pooled (n = 10) and 100 µL was fed to mosquitoes along with cultured *P. falciparum* gametocytes. Counting of *P. falciparum* oocysts was performed in mosquito midguts and the TRA was calculated compared to the group fed gametocytes along with sera from naïve mice. One Way ANOVA and multiple comparison test revealed no difference in the TRA of post-vaccination sera from uninfected versus infected mice (p = 0.985), but significantly greater activity in both groups versus unvaccinated control mice. These results were generated with data from three independent SMFAs: two of them performed with pooled sera from 5 different mice for each experimental group, and the third experiment was performed with 10 pooled sera combined from the two previous experiments. ^*^p < 0.05 compared to naïve group. Data are shown as mean + SEM.

SMFA was performed to evaluate transmission-reducing activity, defined as a reduction in the number of oocysts per mosquito (Fig. 6). Sera from mice that received only Pfs230D1 conferred 36.1% TRA (range 23.1–58.7%) while sera from Hpb-infected and immunized mice conferred 32.8% TRA (range 23.2–37.8%), not significantly different from each other (p = 0.985) but both significantly greater than sera from naïve mice (p = 0.026 and p = 0.042, respectively). Sera from Hpb-infected unvaccinated mice reduced oocyst transmission by 22%, which was not significantly different from the naïve group.

## 4 Discussion

In the current study, we evaluated whether chronic intestinal helminth infection alters the activity of a promising malaria transmission blocking vaccine.

Among the many helminth parasites that elicit immunomodulatory responses, one of the best-studied models is the intestinal nematode *Heligmosomoides polygyrus bakeri* infection in mice [20], [15]. We infected 6-week-old BALB/c mice with the intestinal nematode *H. polygyrus bakeri* (Hpb), and immunized the mice starting 28 days later with Pfs230D1-EPA/Alhydrogel®, a vaccine aimed at reducing transmission of malaria, currently in clinical trials in malaria-endemic areas. Antibody responses to Hpb and the helminth burden in stool were used to assess the establishment of chronic infection. Hpb-infected mice demonstrated marked increase of helminth antigen-specific IgG1, IgA and IgE but not IgG2a antibody responses after 9 weeks of infection, concomitant with an increase of systemic IL-10 and IL-6 levels. This persistent parasite-specific type-2 immune response is normally associated with establishment of chronic infection in primary challenges with Hpb [20], [3], [15].

Vaccine immunogenicity was characterized by production of Pfs230D1-specific activated B cells in the spleen and by antibody titers to Pfs230D1, while vaccine functional activity was assessed by SMFA. Numbers of Pfs230D1-specific B cells, antibody titers and TRA were all significantly higher in vaccinated compared to unvaccinated animals, demonstrating vaccine immunogenicity. Most relevant to this study, production of antigen-specific B cells, antibody titers against Pfs230D1, and TRA did not differ between helminth-infected and uninfected animals.

This study used a submaximal vaccine dosage (∼0.5 µg target antigen) in BALB/c mice based on dose ranging studies conducted in other mouse strains (*manuscript in preparation*), with the expectation that a submaximal response would be more sensitive to immunosuppression. Of note, this vaccine regimen yielded lower TRA levels in BALB/c mice versus levels we have seen for other mouse strains. These differences could be due to strain-specific differences in the immune response, or to the timing of blood sampling (collections on day 7 versus day 14, respectively, after boost). Nevertheless, our data demonstrate that the antibody activity induced by Pfs230D1-EPA was not impaired by Hpb infection, with similar TRA achieved in Hpb-infected and uninfected mice.

Our data collectively demonstrate that the immunogenicity and functional activity of Pfs230D1-EPA/Alhydrogel® vaccine were not impaired by Hpb infection in BALB/c mice. This lends support to the notion that this protein-based malaria transmission blocking vaccine could be implemented in malaria-endemic areas where helminth co-infection is common.

## Author contributions

CHC, JL and PHGG conceived the study. CHC, PHGG, EB, JL, JH, JFU, CA, TBN and PED designed the experiments. CHC, PHGG, JH, EB, NAHA, OM, AM, EK performed experiments. CHC, PHGG, JH, NAHA, OM analyzed data. CHC, PHGG, EB, JH, CA, NAHA, DN, AM, OM, JFU, JL, TBN and PED interpreted the data and wrote the manuscript.

## Competing interests

The authors declare no competing interests.
